# Omega-9 fatty acids: potential roles in inflammation and cancer management

**DOI:** 10.1186/s43141-022-00329-0

**Published:** 2022-03-16

**Authors:** Mohamed A. Farag, Mohamed Z. Gad

**Affiliations:** 1 Pharmacognosy Department, College of Pharmacy, Cairo University, Kasr El Aini St., P.B, Cairo, 11562 Egypt; 2 Department of Biochemistry, Faculty of Pharmacy & Biotechnology, The German University in Cairo, Cairo, Egypt

**Keywords:** MUFA, PUFA, Omega-9, Inflammation; anti-cancer; oleic acid

## Abstract

**Background:**

Omega-9 fatty acids represent one of the main mono-unsaturated fatty acids (MUFA) found in plant and animal sources. They are synthesized endogenously in humans, though not fully compensating all body requirements. Consequently, they are considered as partially essential fatty acids. MUFA represent a healthier alternative to saturated animal fats and have several health benefits, including anti-inflammatory and anti-cancer characters.

**The main body of the abstract:**

This review capitalizes on the major omega-9 pharmacological activities in context of inflammation management for its different natural forms in different dietary sources. The observed anti-inflammatory effects reported for oleic acid (OA), mead acid, and erucic acid were directed to attenuate inflammation in several physiological and pathological conditions such as wound healing and eye inflammation by altering the production of inflammatory mediators, modulating neutrophils infiltration, and altering VEGF effector pathway. OA action mechanisms as anti-tumor agent in different cancer types are compiled for the first time based on its anti- and pro-carcinogenic actions.

**Conclusion:**

We conclude that several pathways are likely to explain the anti-proliferative activity of OA including suppression of migration and proliferation of breast cancer cells, as well stimulation of tumor suppressor genes. Such action mechanisms warrant for further supportive clinical and epidemiological studies to confirm the beneficial outcomes of omega-9 consumption especially over long-term intervention.

## Background

Omega-3, -6, and -9 fatty acids (FAs) are unsaturated fatty acids which impose several biological effects and health benefits. The three omega FAs are generally present in several vegetable oils and in pharmaceutical formulations [1]. Omega-3 and omega-6 FAs are well characterized regarding their benefits to human health [2]. However, omega-9 FAs have recently received wide attention due to emerging studies and discoveries regarding their biological benefits and or risks.

Omega-9 FAs (ω−9 FAs or n−9 FAs) are group of unsaturated FAs that have a double bond in the 9th position from the methyl end. They are either monounsaturated or polyunsaturated. Unlike the 3s and 6s, Omega-9 FAs, they are considered “non-essential” FAs [3].

**Fig. 1:**
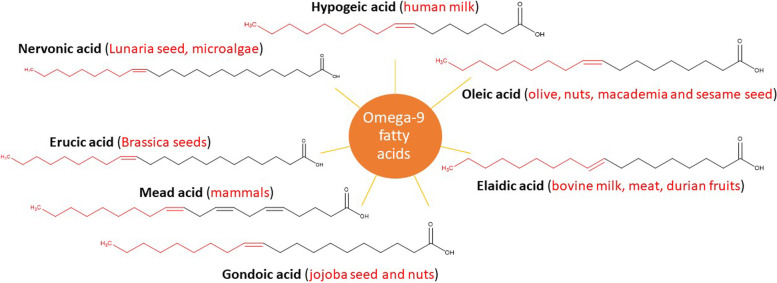
Common omega-9 fatty acids and their natural sources in plant and animals

The most common omega-9 FAs are hypogeic acid (16:1 (n-9), (*Z*)-hexadec-7-enoic acid), oleic acid (18:1 (n-9), (*Z*)-octadec-9-enoic acid), elaidic acid (18:1 (n-9), (*E*,)-octadec-9-enoic acid), gondoic acid (20:1 (n-9), (*Z*)-eicos-11-enoic acid), mead acid (20:3 (n-9), (5*Z*,8*Z*,11*Z*)-eicosa-5,8,11-trienoic acid), erucic acid (22:1 (n−9), (*Z*)-docos-13-enoic acid), nervonic acid (24:1 (n−9), (*Z*)-tetracos-15-enoic acid) (Fig. 1) [3]. Oleic acid received the most attention in research (23,588 citations in PubMed) as compared to hypogeic acid (5 results in PubMed), elaidic acid (516 results in PubMed), gondoic acid (34 results in PubMed), mead acid (145 results in PubMed), erucic acid (699 results in PubMed), and nervonic acid (204 results in PubMed) as searched in April 2021. Oleic acid is the most abundant in many vegetable oils as compared to other omega-9 fatty acids. It represents 40.7% of sesame oil, 17.5% of flaxseed oil, 74.8% of olive oil, 58.8% of rapeseed oil, and 32.7% of pumpkin seed oil.

In general, foods rich in omega-9 FAs include safflower, sunflower, macadamia nut, hazelnut, olive oil, soybean oil, almond butter, avocado oil, and canola oil [4]. Different omega-9 FAs possess diverse pharmacological actions including modulating inflammatory, lipid, cardiovascular (CV) and cancer disorders. This review presents most updated literature on two of the major effects of omega-9 including anti-inflammatory and anti-cancer effects.

## Main text

### Anti-inflammatory and anti-cancer actions of omega-9 fatty acids

#### Oleic acid (OA)

In healthy people, OA is the most abundant FA, found in adipocytes, cell membranes, and plasma [5]. Various animal and plant sources including olive oil, cod oil, corn oil, and palm oil are rich in OA. It has drawn much attention recently due to the widespread use of the Mediterranean food that is wealthy in olive oil, one of the best source of OA among dietary sources [6]. Also, OA is present endogenously as a component of hormones production and cellular membranes [7]. OA is considered a healthier alternative to saturated animal fats and to possess several therapeutic effects. Additionally, OA is used in pharmaceutical industry as a solubilizing agent or emulsifier [8, 9].

### Anti-inflammatory actions

OA-rich diet has positive outcomes in inflammatory-related disorders. It modulates immune system by activation of various immune competent cell pathways [10]. However, controversial data exist in literature regarding its biological value in different cellular functions. Here are some positive studies that show the anti-inflammatory actions of OA in several organs systems.

### Eye inflammation

OA possesses an anti-inflammatory effect against hyperlipidemia-induced retinal inflammation in male Wistar rats. High OA diet (17.5% olive oil rich diet) administered for 90 days lowered the levels of the proinflammatory serum and retinal cytokines like IL-1-β, TNF-α and MCP-1. It also decreased the expression of serum C reactive protein (CRP), serum pro-inflammatory eicosanoids (LTC4, LTB4, and PGE2), and retinal expression of BLT-1, EP-4, EP-1, and COX-2 compared to control rats fed with 7.0% lard rich diet [10, 11]. It has been demonstrated that OA have possible therapeutic benefits in enhancing both hydrophilic and lipophilic compound ocular drug delivery [12]. Moreover, several studies have indicated that lipid-based lubricants can help relieve some symptoms of dry eye [13]. Thus, we believe, due to its inflammatory actions, enhancing drug delivery and improving dry eye effects, OA addition to topical ophthalmic preparations is worth to be extensively studied in certain eye disorders.

### Skin inflammation

OA was shown to alleviate skin inflammation by altering neutrophils’ role in immunity; however, binding to albumin diminishes its anti-inflammatory activity. A study investigated the effect of incorporation of OA within nano-structured lipid carriers (OA-NLC) in improving the anti-inflammatory actions. Results showed that in the presence of albumin, the OA-NLC, in contrast to unconjugated free OA, aborted elastase release and superoxide generation, and suggestive for the improved nano-formulation impact on OA anti-inflammatory effect that has yet to be reported for other functions. Topical application of OA-NLC as an ointment alleviated neutrophil infiltration and relieved skin inflammation severity [7, 14]. Whether such disease improvement is correlated with increased OA levels inside the skin tissue should be determined to be more conclusive.

Additionally, OA plays a crucial role in wound healing by inducing rapid wound closure which is essential to prevent superimposing infections and delayed healing. Cutaneous application of OA on wounds in Wistar rats resulted in accelerated proliferative stage, regeneration of epithelial cells, and proper collagen and keratin formation. In addition, neovascularization was enhanced in the initial inflammatory phase because of an increase in vascular endothelial growth factor (VEGF) expression, which exhibits an essential function in the angiogenesis process [15]. Another report by Rodrigues et al. indicated that OA enhances NF-κB and TNF-α secretion after 1 h of wound formation in rats. However, a decrease in IL-6, IL-1, and MIP-3α levels, and NF-κB release, was observed 24 h after wounding suggesting that OA hastens wound healing's inflammatory processes [14].

**Fig. 2:**
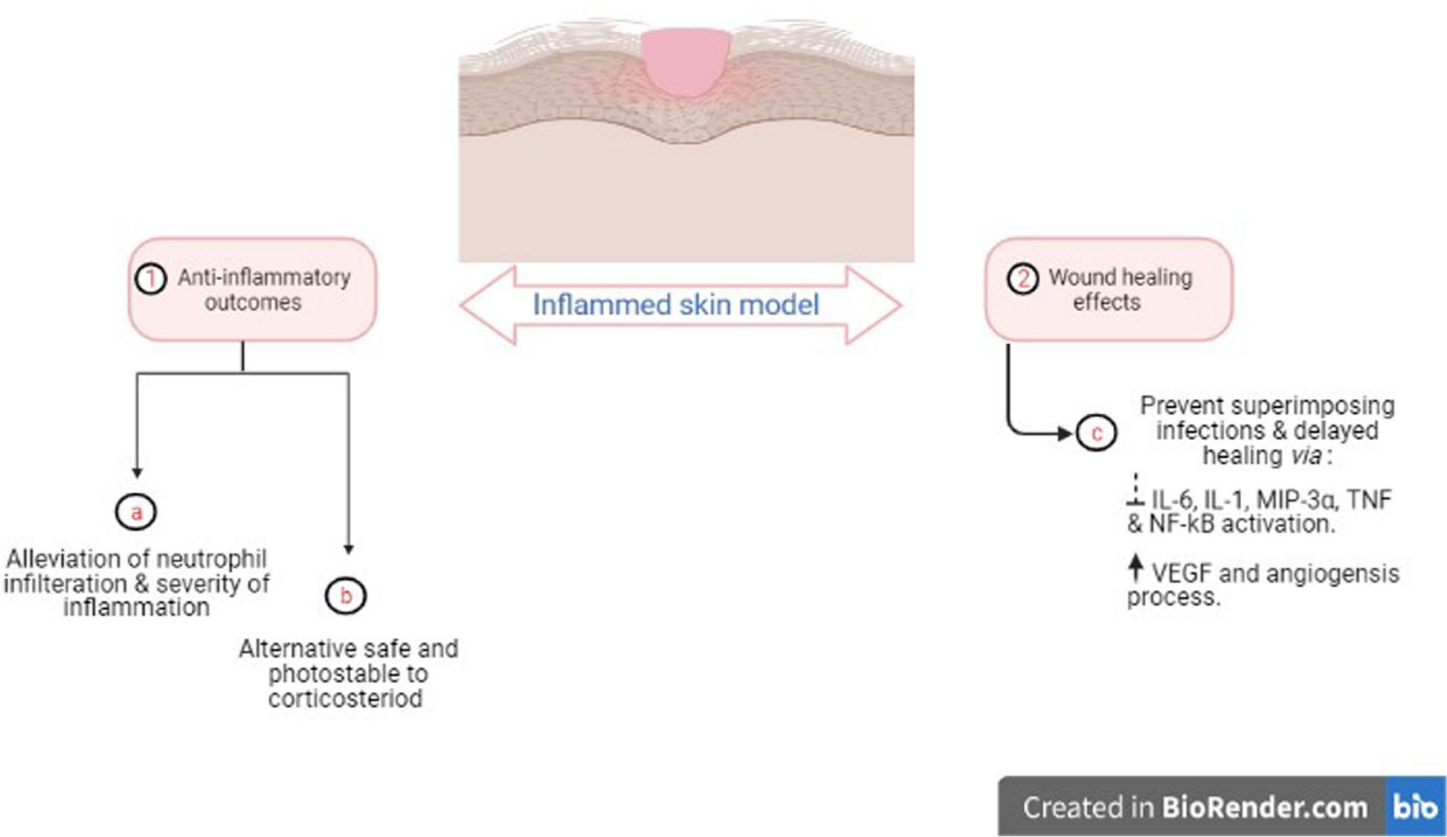
Summary of the action mechanisms of oleic acid (OA) in modulating skin inflammation and wound healing effects

Further studies on the anti-inflammatory action of OA in skin included a study by [16]. The study determined the potential of OA to act as an alternative to corticosteroids for the treatment of UV-induced skin inflammation. OA was added to semisolid preparations based on Lanette® or Pemulen® TR2. Both formulations reduced ear edema in mice after repeated treatments at 24, 48, and 72 h after UVB exposure. The anti-inflammatory property appeared to be mediated by glucocorticoid receptors. The authors suggested the use of OA as a promising alternative to glucocorticoids given that OA is safe and not photosensitive even at relatively high dose (13%) [16]. Figure 2 summarizes the postulated effects of OA on skin inflammation.

### Lung inflammation

Pneumonitis is a general term that refers to lung tissue inflammation. Physicians commonly use the term pneumonitis to refer to noninfectious causes of lung inflammation. Lung inflammation may be acute or chronic, and there are a variety of causes, including environmental factors, infections, and diseases such as asthma and bronchitis [17]. Lung damage caused by OA is a common used model that closely resembles human disease [18]. However, OA has been found to possess anti-inflammatory activities towards activated neutrophils. OA-based nanosystems mitigated against acute respiratory distress syndrome in mice via the suppression of neutrophils [19].

### Liver inflammation

When a disease-causing microbe or drug attacks liver cells, liver inflammation occurs. The term hepatitis refers to liver inflammation. Hepatitis is mainly caused by a viral infection, but it may also be caused by an autoimmune disorder. Alcohol, toxins, and some medications can also cause liver damage, which can lead to inflammation. Hepatitis may also be caused by hereditary disorders, as well as a chronic obstruction of bile flow. The type of hepatitis determines the severity, treatment, and outcome of the liver inflammation. Fibrosis, cirrhosis, and hepatocellular carcinoma may all be caused by chronic liver inflammation [20].

Extra virgin olive oil, rich in OA, can help prevent inflammation, mitochondrial dysfunction, insulin resistance, endoplasmic reticulum (ER) stress, and oxidative stress by activating various signaling pathways in hepatic parenchymal cells. The following are the most important pathways that lead to the resolution or prevention of liver injury: (1) induction of the nuclear factor erythroid 2-related factor 2 (Nfr2), leading to antioxidant signals; (2) suppression of (NF-κB), which prevents the cellular inflammation response; and (3) suppression of the PERK signaling pathway that results in prevention of autophagy, ER stress, and lipogenic response [21]. OA also reduces hepatocellular lipotoxicity caused by palmitic acid by inhibiting pyroptosis and ER stress [22].

### Sepsis

Sepsis is a potentially fatal disease, which occurs when the body’s immune system starts to attack its own tissues in response to an infection. Septic shock may develop from sepsis. This dangerously low blood pressure can lead to organ failure and death. While any infection—viral, bacterial, or fungal—may cause sepsis, infections of the lungs, urinary tract and digestive systems, blood (bacteremia), and skin (catheter sites, burns or wounds) are the most common causes [23]. In a study by Medeiros-de-Moraes et al., mice developed sepsis using cecal ligation and puncture (CLP) model and upon treatment for 14 days with omega-9 elevated levels of the anti-inflammatory IL-10 concurrent with reduction of proinflammatory IL-1β and TNF-α levels in fluid of septic animals. Furthermore, omega-9 intake reduced systemic levels of corticosterone. According to the authors, omega-9 can play a positive anti-inflammatory role in sepsis by reducing leukocyte influx and rolling, neutralizing cytokine output, and regulating bacterial growth through a PPAR-γ signaling pathway [24].

In another study, OA pretreatment for 14 days improved longevity, prevented kidney and liver damage, and reduced plasma levels of NEFA in mice exposed to CLP sepsis model. OA intake also decreased reactive oxygen species (ROS) and increased 5' AMP-activated protein kinase (AMPK), carnitine palmitoyltransferase IA (CPT1A), and uncoupling protein 2 (UCP2) levels [25].

### Ulcerative colitis and intestinal inflammation

Ulcerative colitis (UC) is an inflammatory bowel disorder that causes ulcers and inflammation in the gastrointestinal tract. UC occurs when the colon or/and rectum lining becomes inflamed. Genetic predisposition, dysregulated immune responses, defects in epithelial barrier, and environmental factors all contribute in the pathogenesis of that disease. Despite the fact that there is no cure, medication can significantly reduce signs and symptoms of the disease and can lead to long-term remission [26].

**Fig. 3:**
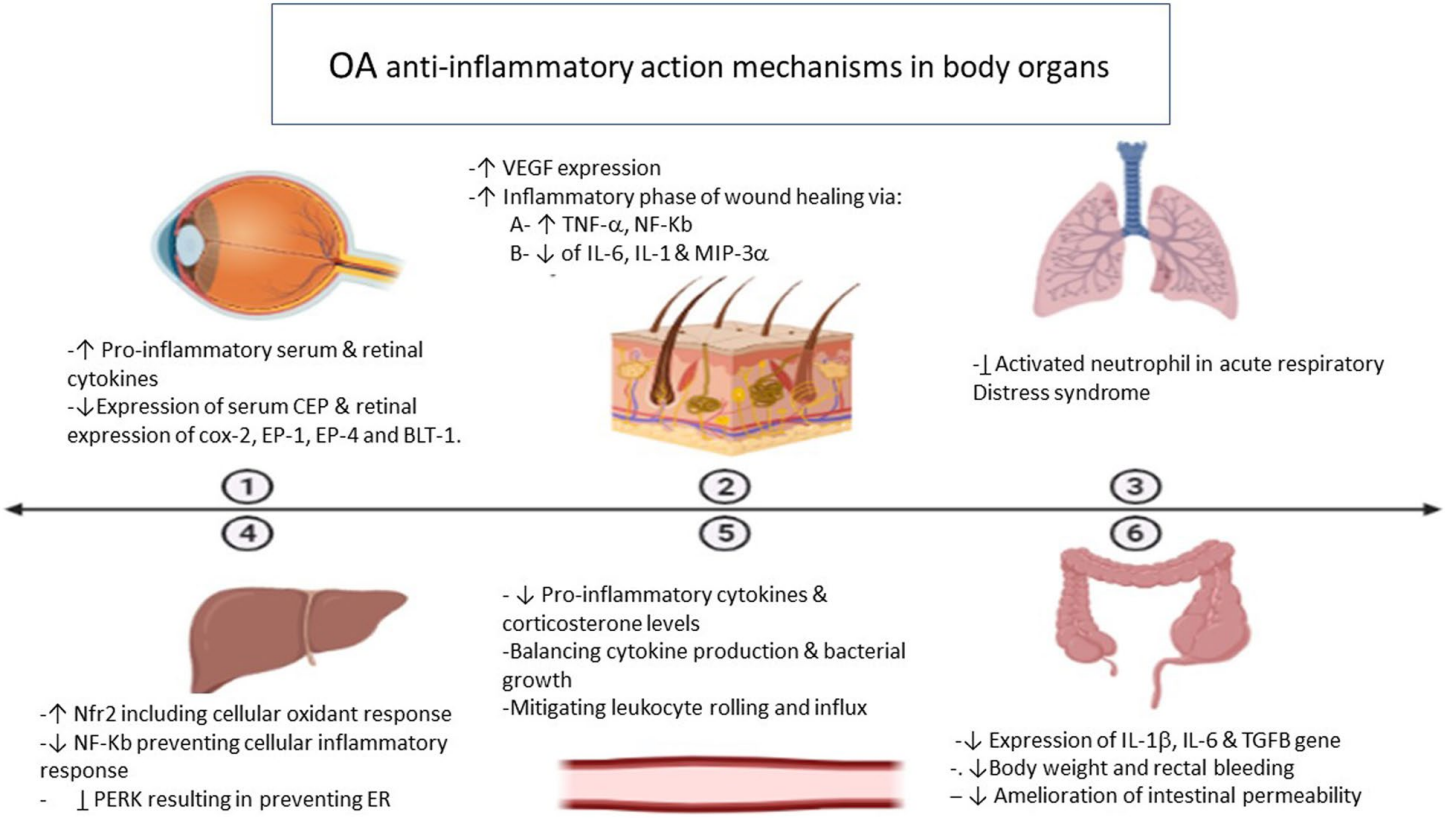
Oleic acid (OA) anti-inflammatory action mechanisms in the different body organs viz. eye, skin, lung, liver, blood vessels, and intestine

In a trial to reduce the burden of meat products diet in cases of ulcerative colitis, Fernández et al. fed experimentally induced UC rats with acorn-fed ham rich in OA. The gut microbiota was altered because of the diet, with significant increase in bacterial genera having anti-inflammatory properties (Alistipes, Blautia, Dorea, Parabacteroides). It also had a powerful anti-inflammatory effect, which helped to avoid UC symptoms including macroscopic score of colitis, disease activity index, density of inflammatory cell in colon, epithelium alteration in colon mucosa, proinflammatory IFN-γ and IL-17 levels, and myeloperoxidase titers in colon as compared to rats fed conventional vegetable diet [27]. In another report, Cariello et al. examined whether extra-virgin olive oil (EVO) intake is able to exert a prophylactic role in a dextran sodium sulfate (DSS) colitis-mediated animal model. Results revealed that EVO administration reduced the rectal bleeding, loss in body weight, and TGFβ, IL-1β, and IL-6 levels. It also reduced intestinal permeability and inflammation-related histopathological features [28]. A summary of OA anti-inflammatory actions in different organs is depicted in Fig. 3.

### Insulin resistance (IR) and type 2 diabetes mellitus (T2DM)

Among the main factors involved in the development and activation of IR and T2DM is mitochondrial dysfunction that may lead to inefficient fatty acid oxidation (FAO). OA enhanced FAO genes expression by PGC1α deacetylation through PKA-dependent stimulations of SIRT1-PGC1α complex. The anti-inflammatory actions of OA also included lowering the expression of the inflammatory mediators; E-selectin and sICAM, upregulating free fatty acid receptor-4 (FFAR4), promoting M2 expression, decreasing phosphate and tensin homolog (PTEN), increasing adiponectin, and downregulating protein phosphatase 2A (PP2A). OA also controlled IR and T2DM by improving β cell function and endothelial functions, oxidative stress, hypothalamic function, glucolipotoxicity, apoptosis, and dysregulation of enzymes [29]. Whether administration of OA with anti-diabetic agents like metformin could lead to a synergized action has yet to be determined.

### Anti-cancer actions

OA has been shown in numerous reports to inhibit cellular proliferation in several tumor cell lines. OA inhibited HER2 overexpression, a well-known oncogene involved in the development, and metastasis of numerous human cancers. In carcinoma cells, OA also plays a significant role in the intracellular calcium signaling pathways related to apoptosis and growth induction. The mechanisms underlying the apoptotic event caused by OA are linked to the rise in intracellular caspase 3 activity and the development of ROS [30].

OA downregulated cancer activity of human esophageal cells (HEC) through several mechanisms including suppressing cell proliferation, and cellular migration and adhesive properties as mediated via activating tumor suppressor genes (p27, p21 and p53). Although OA treatment of HEC did not influence the number of colonies, it inhibited the colony size remarkably. Further, OA is recognized for its anti-proliferative effect in other types of cancer including colorectal cancer, where OA induced apoptosis as well as breast cancer by regulating HER2 gene expression [6]. Nevertheless, more studies using in vivo animal grafted models should be performed to confirm OA anti-tumor actions.

Jiang et al. explored OA anti-cancer properties and mechanisms in tongue squamous cell carcinoma (TSCC). Results revealed that OA efficiently suppressed proliferation of TSCC cells. It markedly promoted cell cycle G0/G1 arrest, decreased Bcl-2 and Cyclin D1 expression, and elevated the proportion of apoptotic cells, concurrent with increased p53 expression and caspase-3 cleavage. OA also caused autolysosome formation and decreased p62 expression as well as LC3 I/LC3 II ratio. Furthermore, post-OA therapy, expression of p-mTOR, p-Akt, p-4E-BP1, p-S6K, and p-ERK1/2, was dramatically reduced in TSCC cells. It was concluded from the study that OA possessed an anti-cancer activity in TSCC through enhancing autophagy and apoptosis via inhibiting the Akt/mTOR signaling pathway [31].

Proteins such as α-lactalbumin and lactoferrins are among the other macromolecules with which OA interacts and mediates its anti-cancer properties. In patients with advanced cancer, a combination of OA and Gc protein-derived macrophage activating factor (GcMAF) was shown to have significant influence on immune system activation and decrease of tumor mass [32]. The release of nitric oxide is linked to the GcMAF stimulating effects on macrophages. Furthermore, OA increased the efficacy of cancer drugs in a synergistic manner. For instance, OA increased the potency of Herceptin, a breast cancer drug that targets the HER-2/neu gene [33]. In addition, n-butyl and phenyl derivatives of OA exhibited great growth inhibitory activity in human HT-29 colon and MCF-7 breast cell lines [34]. Synthesis of other OA analogues could help identify even more active anti-tumor agents based on OA using either in silico drug modelling or combinatorial chemistry.

**Fig. 4:**
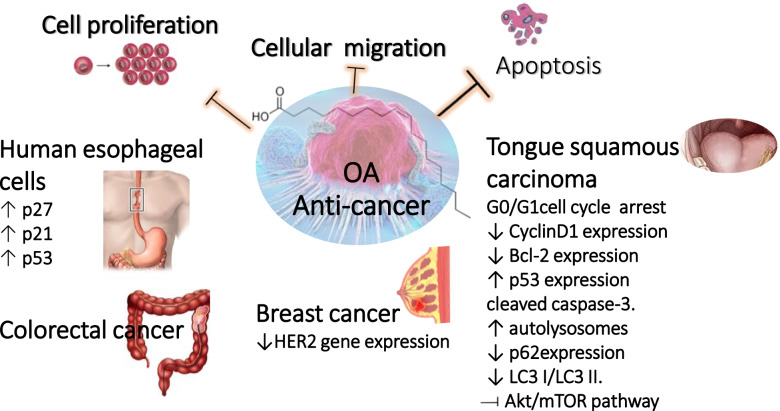
Summary of oleic acid (OA) action mechanisms as anti-tumor agent in different cancer types

A summary of OA anti-cancer effects and action mechanisms in different cancer types is depicted in Fig. 4.

#### Elaidic acid (EA)

Elaidic acid, trans-isomer of oleic acid, has received recently wide consideration being a common trans-fat, which has been linked to heart disease. EA occurs naturally in bovine and caprine milk (around 0.1% of the FAs) [35], some meats, in addition to plant sources such as durian fruits.

Studies in human have found a connection between the usage of industrial trans-fatty acids (TFAs), such as EA, and the incidence of cardiovascular diseases, prompting many countries to pass legislation prohibiting the use of industrial TFAs in food. However, TFA cannot be totally removed from human food because they are naturally found in dairy and meat derived from ruminant animals [35].

Da Silva et al. assessed the anti-inflammatory properties of several TFAs, including EA in several cell lines including (HUVEC) and (HepG2) cells for 24 h at concentrations ranging from 5-150 μM. Stearoyl-CoA desaturase (SCD-1), a main enzyme in FAs biosynthesis, expression increased after EA was added. EA additionally decreased expression of inflammatory genes in HUVEC cells, but not HepG2 cells [36], suggestive for a selective effect against cancer cell lines.

In general, administration of TFA, including EA, represents health hazards in many studies. Wang et al. reported that higher circulating EA is connected with increased long-term morbidity and mortality in the general population [17]. Li et al. presented same conclusion, reporting that plasma EA levels are linked to an elevated risk of CVD mortality [37].

EA also induced cholesterogenesis in Hepa1-6 hepatoma cells in vitro by activating the sterol regulatory element-binding protein (SREBP) cleavage-activating protein (SCAP), mostly by reducing intracellular free cholesterol and cholesterol-dependent SCAP repression. The increase in liver cholesterol and non-alcoholic fatty liver disease (NAFLD) caused by industrial TFA may be attributed to this pathway. In contrast to cis-unsaturated and saturated diets, EA increased liver/gonadal fat mass ratio, hepatic cholesterol content, alanine aminotransferase activity, steatosis, and markers of fibrosis in mice, implying increased NAFLD [38]. This provided further evidence that industrial TFA can cause liver damage, primarily by initiating fatty liver that develop into fibrosis.

In contrast to cis-unsaturated fatty acids, the cancer-promoting properties of TFAs have been well documented. Kishi et al. investigated EA’s effects and signaling in cells of colorectal cancer (CRC). Oral consumption of EA to mice increased the metastasis of HT29 human CRC cells, which were inserted into the mice scapular subcutaneous tissue. EA was incorporated into the plasma membrane's cholesterol rafts that embraced epidermal growth factor receptors (EGFR). In HT29 cells, EA elevated nanog and c-myc levels, while decreasing PGC-1A levels *via* lipid raft-linked EGFR signaling [39]. Ohmori et al. also demonstrated the enhancement of survival, growth, and invasion of the colorectal cancer cell lines, HT29, and CT26 by EA [40].

Other negative effect of EA included its neurotoxicity. Following treatment of SH-SY5Y neuroblastoma cells with various levels of EA (10, 20, 50, 100, 200, 400, and 800 μM) for 24 h at 37 ^o^C, suppression of cell viability, elevation of cell apoptosis, and loss of mitochondrial membrane potential (MMP) were observed. Furthermore, EA caused significant changes in cellular redox status. Higher doses of EA (200, 400, or 800 μM) increased the production of ROS, including lipid peroxide and malondialdehyde levels, upregulated Nrf-2, and downregulated heme oxygenase 1 (HO-1), two primary anti-oxidative players. These results inferred that EA suppressed SH SY5Y cell growth and enhanced apoptosis by increasing oxidative stress and triggering the ER stress/UPR signaling pathway as well as the GRP78/ATF4/CHOP pathway [41]. Whether examined doses of elaidic acid in these studies are comparable to its level in natural resources should be made to be more conclusive for these effects.

#### Gondoic acid (GA)

GA, cis-11-Eicosenoic acid, is found in a variety of plant oils and nuts; in particular jojoba oil [42]. It is also found in red cell membrane with upsurge levels in children with regressive autism. No studies were found regarding the health effects of this particular omega-9 fatty acid and warranting for future work to verify its health benefits or toxicity.

#### Mead acid (MA)

Mead acid is formed de novo in animals derived from OA. Its elevated level in blood is indicative of “essential” fatty acid (EFA) deficiency [43]. MA’s functions in normal physiological conditions had not been thoroughly studied. Increased metabolism of oleic acid to MA occurs in the absence of adequate α-linolenic acid and linoleic acid [44]. MA levels in chicken and human infant cartilage have long been recognized to be high, even in the absence of EFA deficiency. It was proposed that MA prevents cartilage calcification, and also to occur in avascular tissues like the cornea and lens [45].

With regard to the anti-inflammatory features of MA, Yoshida et al. investigated the impact of MA-enriched diet on experimentally induced bowel lesions. Rats were given either an MA-enriched or a standard diet. Acute bowel lesions were induced, after 7 days of feeding, by injecting 10 mg/kg indomethacin subcutaneously. Results indicated that dietary supplementation of MA (5% *M. alpine* oil-enriched diet; *M. alpine* oil contains 17% MA) had both therapeutic and prophylactic actions on experimentally induced bowel lesions [46]. In a different aspect, simultaneous addition of MA as adjunct treatment with aggregating agents to platelets enhanced platelets’ response [47].

Concerning the anti-cancer properties of MA, Kinoshita et al. explored the impact of MA on the production and proliferation of *N*-methyl-*N*-nitrosourea (MNU)-induced mammary carcinoma in rats. All mammary tumors were found to be luminal A carcinoma. The MA-containing diet dramatically inhibited the development and progression of mammary carcinogenesis via the suppression of cell proliferation [48]. MA also inhibited the growth of KPL-1 human breast carcinoma both in vivo and in vitro, but had no effect on angiogenesis. VEGF signaling to tumor cells was one of the proposed mechanisms of action [49]. Furthermore, MA inhibited some pro-cancerous properties in three different human cell lines: MCF-7 from breast, T-24 from urothelium, and HRT-18 from colon (Heyd and Eynard, 2003).

#### Erucic acid

Erucic acid is mostly abundant in Brassica seeds (*Eruca sativa*), known as arugula (USA) or rocket (UK), in addition to Indian mustard (*Brassica juncea*) and rapeseed (*Brassica napus*) [50].

Erucic acid is a PPAR-δ ligand, found to lead to improved cognitive parameters in animal models through its anti-oxidative and anti-inflammatory actions [51]. It may also act as a neuroprotective, anti-tumor, and myelin protective agent in Parkinson’s disease, glioblastoma, and neuroblastoma [52]. Its remyelinating property might also be valuable in the management of multiple sclerosis [52]. The suppression of p38 MAPK and NF-B signaling are thought to be the molecular mechanisms through which erucic acid exerts its anti-inflammatory properties (Liang et al. 2020) [53].

Regarding the anti-tumor effect, erucic acid is present abundantly in the Chinese diet and suggestive that the low brain cancer frequency in children among the Chinese population is credited to high content of erucic acid in Chinese women breast milk [54]. Erucic acid is believed to possess an anti-tumor activity especially in C6 glioblastoma, where erucic acid suppressed cellular proliferation by blocking synthesis of DNA (arresting the cell cycle at S-phase) as well as direct cellular interaction [51]. The anti-proliferative action of erucic acid on the glioblastoma cells is related to its agonistic effect on peroxisome proliferator-activated receptors (PPARs). Additionally, co-administration of erucic acid with doxorubicin alleviated the cardio toxic and hepatotoxic effects of doxorubicin and with doxorubicin better tolerated with enhanced anti-cancer effect [55]. Nevertheless, erucic acid has been shown to induce myocardial lipidosis, hepatic steatosis, and cardiac lesions in animals. These effects limit its inclusion in edible oils by regulatory agencies [56] and reduce its clinical applications.

#### Nervonic acid (NA)

NA is (Z)-15-tetracosenoic acid, 24:1 (n9) occurs naturally as an extension product of oleic acid, with erucic acid as the immediate precursor. NA is abundant in peripheral nervous tissue and in the animal brains white matter, where nervonyl sphingolipids are abundant in nerve fiber myelin sheath [52]. NA has received much attention owing to its close association with brain development. NA levels of human brain sphingolipids rise dramatically from birth to a peak at 4 years, and to remain constant afterwards [57]. Natural resources of NA include oil crop seeds especially seed oils of *Lunaria* species and oil-producing microalgae [58]. NA is required for the formation of brain myelin and can be used as a marker of brain maturity. Infants’ neurodevelopment can be aided by formula feeding, and customers and producers may benefit from a healthier alternative to milk powder. However, the contradictory findings regarding NA contents of patients with psychosis, depressive disorders, Alzheimer’s disease, and cardiovascular disease warrant further study.

In a study by Lewkowicz et al. on lipids profiling in an induced model of brain autoimmune encephalomyelitis, it was shown that during acute inflammation, NA biosynthesis was downregulated as a result of shifting lipids metabolism pathways of common substrates into pro-inflammatory arachidonic acid formation [59]. NA has been shown to alleviate Parkinson’s disease-related tremors and multiple sclerosis-related numbness. It can also be used to treat schizophrenia and to alleviate the symptoms of Alzheimer's disease in the early stages [58].

#### Hypogeic acid (HA)

Hypogeic acid is found in human milk. Very few studies involving HA biological effects have been found in the literature. In one study by Astudillo et al., the anti-inflammatory properties of hypogeic acid along with palmitoleic acid were investigated. The majority of hexadecenoic fatty acids were esterified in a specific pattern, palmitic acid at sn-1 location and hexadecenoic acid at sn-2 as revealed in mouse peritoneal macrophages. When macrophages are stimulated with inflammatory stimuli, this species decreases dramatically. Although the majority of the released hexadecenoic acid appeared as a free FA, a large portion is moved to other phospholipids to construct hexadecenoic acid-containing inositol phospholipids that are further recruited to yield fatty acid esters of possible anti-inflammatory properties [60]. The findings suggest that conversion of these fatty acids to other lipid mediators can account for some of their anti-inflammatory activity. HA has been proposed as a potential marker for the development of foamy cells in atherosclerosis, but whether such marker is a reactive homeostasis response or indicative of disease progression should be clarified.

## Conclusions

Omega-9 fatty acids exhibit essential myriad of pharmacological activities that pose them as potential candidate to alleviate many pathological conditions. The observed anti-inflammatory effects reported for oleic acid, mead acid, and erucic acid were directed to attenuate inflammation in several physiological and pathological conditions such as wound healing and eye inflammation by altering the production of inflammatory mediators, modulating neutrophils infiltration, and altering VEGF effector pathway.

The anti-neoplastic action of omega-9 fatty acids is though controversial compared to its anti-inflammatory actions, with the effect varies with the type of cancerous tissue and the effector pathway. Most documented anti-neoplastic action of omega-9 is evidenced in case of olive oil-rich diets. These diets enriched in oleic acid content are believed to possess chemo preventive effect against breast cancer. Oleic acid anti-tumor action is mediated via multiple mechanisms including suppression of proliferation and migration and breast cancer cells, as well activation of tumor suppressor genes.

On the other hand, several pathways are believed to explain the proliferative activity of OA. In MCF-7 and MDA-MB-231 breast carcinoma lines, OA enhanced metastasis and cellular proliferation through activation of free fatty acid receptor 1 (FFAR1) and FFAR4 receptors that are activated by medium-chain FAs like OA. Such receptors are also present on the surface of other cells such as insulin secreting in pancreatic β cells and adipose tissue, and upon activation, they alter the intracellular Ca^2+^ concentration. In addition, OA activates AKT pathway (protein kinase B) where AKT2 induces production of Integrin B that facilitates invasion along with AKT-1 activation that promotes cell migration as well along with epidermal growth factor receptor (EGFR) OA-dependent activation [61].

**Fig. 5:**
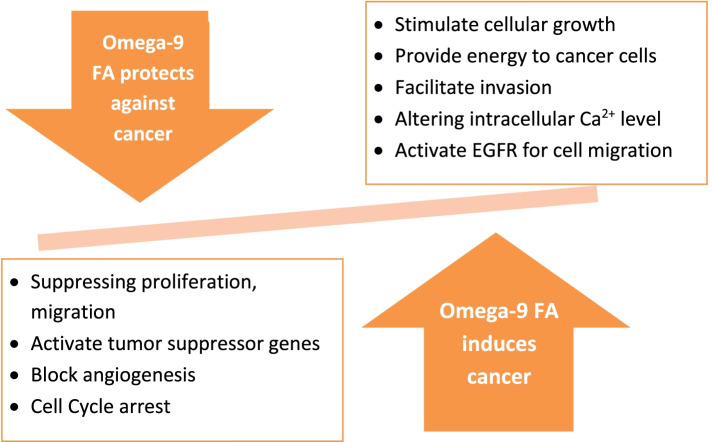
Anti- or pro-cancer actions of omega-9 fatty acids

In general, reports on the anti-inflammatory and anti-cancer actions of omega-9 FAs, other than oleic acid, are quite scarce especially for the clinical studies. Further research is warranted to provide more conclusive data about their prophylactic and therapeutic value in those two widespread disorders. Figure 5 summarizes the pro- and anti-cancer actions of omega-9 FAs. These results pose omega-9 FAs as promising therapeutic agents warranted to be further studied in different pathological conditions, especially in inflammation and cancer. These effects have yet to be confirmed though using randomized controlled trials to reveal solid conclusive evidence for comparative actions using isolated oils enriched in certain omega-9 fatty acids or better using individual fatty acids. Execution of more epidemiological studies with more advanced methodologies (lipidomics, metabolomics, and molecular techniques) will yield more reliable information about omega-9 action mechanisms at different cellular levels and provide the optimum ratio of these different fatty acids to be recommended for dietary fat intake. How gut microbiota interact with omega-9 to mediate for its anti-inflammatory effects if any is also an area less explored and should be considered using ex vivo or ideally in vivo assays. Modification of these fatty acids using different techniques, i.e., nanoformulation, nanoemulsion, and encapsulation, should also help improve their bioavailability and ultimate biological effects.

## Data Availability

Not applicable.

## Abbreviations

FAs: Fatty acids
ω−9 FAs: Omega-9 FAs
OA: Oleic acid
IR: Insulin resistance
T2DM: Type 2 diabetes mellitus
TSCC: Tongue squamous cell carcinoma
EA: Elaidic acid
SREBP: Sterol regulatory element-binding protein
SCAP: Cleavage-activating protein
NAFLD: Non-alcoholic fatty liver disease
TFAs: Trans-fatty acids
SCD-1: Stearoyl-CoA desaturase
CRC: Colorectal cancer
GA: Gondoic acid
MA: Mead acid
NA: Nervonic acid
HA: Hypogeic acid
FFAR1: Free fatty acid receptor 1
EGFR: Epidermal growth factor receptor
